# Effects of dietary black cumin (*Nigella sativa* L.) meal on performance, blood metabolites, and digestibility in a rice straw-based diet of fattening Garut lambs

**DOI:** 10.14202/vetworld.2024.2152-2158

**Published:** 2024-09-25

**Authors:** Diky Ramdani, Karina Natasya Juandita, Iman Hernaman, Ken Ratu Gharizah Alhuur

**Affiliations:** 1 Department of Animal Production, Faculty of Animal Husbandry, Universitas Padjadjaran, Sumedang, 45363, Indonesia; 2 Postgraduate Study Program, Faculty of Animal Husbandry, Universitas Padjadjaran, Sumedang, 45363, Indonesia; 3 Department of Animal Nutrition and Feed Technology, Faculty of Animal Husbandry, Universitas Padjadjaran, Sumedang, 45363, Indonesia

**Keywords:** black cumin meal, blood metabolites, digestibility, lamb, performance, and rice straw

## Abstract

**Background and Aim:**

During black cumin oil production, black cumin meal (BCM) is produced as a by-product. This study investigated the potential use of BCM to partly replace concentrate in a rice straw-based diet of fattening Garut lambs.

**Materials and Methods:**

Twenty-eight heads of male Garut lambs aged approximately10 months with an average initial body weight of 20.7 kg/head (coefficient of variation 12.9%) were used. A completely randomized design was used to compare four different levels (0% [control], 5% [BCM-5], 10% [BCM-10], and 15% [BCM-15]) of BCM in rice straw- and concentrate-based diets on the performance of Garut lambs during 35- and 70-day feeding trials using seven replicates (n = 7). Blood metabolites and nutrient digestibility were also measured after the performance study.

**Results:**

BCM was rich in crude protein (36.8%) and tannins (21.6%). The BCM-10 and BCM-15 treatments increased (p < 0.05) average daily gain and feed efficiency compared with the control treatment in the 35-day feeding trial. All BCM treatments had greater dry matter (DM) intake compared with the Control at 70 days (p < 0.05). Furthermore, BCM-10 and BCM-15 had greater (p < 0.05) total protein, DM, and organic matter (OM) digestibility. BCM-15 had the highest (p < 0.01) blood triglyceride while BCM-10 tended to have higher (p < 0.1) blood glucose concentrations among the other treatments.

**Conclusion:**

BCM supplementation can partly replace concentrate and improve the overall quality of rice straw and concentrate-based diets, resulting in improved performance of fattening Garut lambs due to increased DM and OM digestibility, as well as protein and energy absorption. Approximately 10% of the BCM supplementation is suggested as the optimum level.

## Introduction

Sheep farming can improve community wealth in rural areas of developing countries, such as Indonesia. The Ministry of Agriculture [1] reported that over 14.063 million heads of sheep are well-suited to various agroecological regions in Indonesia. However, the massive conversion of farming lands into houses and industries has decreased the availability of high-quality forage to feed ruminants. Rice straw is the only available and affordable roughage, as most Indonesians consume rice as a main staple. Unfortunately, rice straw is low quality and fibrous forage, less palatable, and not well digested in the rumen due to its large contents of lignin and silica [2]. Farmers often prefer rice straw as their primary crop’s roughage because it is readily accessible and affordable [3]. In this case, high-quality feed supplements, such as concentrates, are greatly required to improve the overall quality of a sheep diet, especially during the fattening phase [4]. Most farmers buy formulated concentrates mixed in local commercial feed mills. However, the price of mixed concentrates increases over time following increases in the proportion of most feed ingredients. Feeding management is a key factor in a successful sheep farm business because it accounts for up to 80% of the total cost of animal production [5]. Thus, alternatively, high-quality and affordable feed ingredients must not only increase feed efficiency, but also reduce production costs.

Black cumin (*Nigella sativa* L.) is a herb that grows across a vast region spanning the Eastern Mediterranean, Northern Africa, the Indian subcontinent, and South-west Asia. It is grown in numerous countries such as Egypt, Iran, Greece, Syria, Albania, Turkey, Saudi Arabia, India, and Pakistan. Recognized as a cure-all, black cumin seed in various forms, such as essential oil, paste, powder, and extract has traditionally been used in medicine for a variety of diseases [6]. This herb has also attracted increasing attention from pharmaceutical sectors, researchers, and stakeholders in agriculture. Black cumin essential oil is extracted from the seeds and widely used in human medicine. Black cumin meal (BCM) is also produced as a by-product during oil extraction. BCM contains high levels of crude protein (CP) and bioactive compounds such as thymoquinone that can function as antioxidants and a sheep immunity booster [7]. In another study, Paarakh [8] reported that the bioactive compounds in BCM are tannins and flavonoids. Tannins and flavonoids are polyphenolic constituents. Based on a systematic review study, Ramdani *et al*. [9] concluded that polyphenols have the potential as natural dietary additives and anthelmintics to improve the production and health performance of ruminants. However, their efficacy may vary depending on the plant sources, extraction methods, amounts, and diets in various ruminant studies conducted in different situations.

High-protein and bioactive substances in BCM are expected to improve the quality of sheep diets. However, the use of BCM as a feed supplement to partly replace concentrate and improve the overall quality of a rice straw-based diet of fattening Garut lambs is not yet globally popular, including in Indonesia. A meta-analysis reported that increasing BCM supplementation linearly increased average daily gain (ADG) and dry matter intake (DMI) [10].

Therefore, this study tested the hypothesis that BCM supplementation can partly replace mixed concentrates and improve the overall quality of rice straw-based diets, leading to improved growth performance, nutrient digestibility, and blood metabolite parameters in intensive fattening of Garut lambs.

## Materials and Methods

### Ethical approval

This study was approved by the Research Ethics Commission of Universitas Padjadjaran Number: 246/UN6.KEP/EC/2023.

### Study period and location

This study was conducted from March to June 2023 at research, teaching, and laboratory facilities within the Faculty of Animal Husbandry, Universitas Padjadjaran, Jatinangor campus, West Java, Indonesia. The average daily temperature and humidity during the experiment were 21.8°C and 79.8% relative humidity (RH) (morning); 32.9°C and 48.9% RH (afternoon); 28°C and 65.63% RH (evening).

### Animals and experimental procedures

Twenty-eight heads of growing lambs of Garut breed (Decree of Indonesian Agricultural Minister No. 2914/Kpts/OT.140/6/2011) were used in this study at approximately 10 months old with an average initial body weight (BW) of 20.7 kg/head (coefficient of variation 12.9%). All lambs were grouped into four groups, with seven replicates per group. Each lamb was housed in an individual pen (1.0 m long × 1.0 m width × 1.0 height). The experimental period was 84 days, of which the first 14 days were used as an adaptation period, followed by a 70-day feeding trial. During the adaptation period, each lamb was cleaned, sheared, and treated with an oral anthelmintic (1 mL/head, Veta Bendazole®, PT. Sarana Veterinaria Jaya Abadi, Indonesia). B complex vitamin injection (5 mL/head, B-Sanplex®, PT. Sanbe Farma, Indonesia), and antibiotic injection (2.5 mL/head, Limoxin-200 LA®, Interchemie Werken BV., Holland).

Each experimental diet was offered to the experimental lambs 3 times a day, at 07.00 am, 12.00 pm, and 04.00 pm (Jakarta time). Each experimental diet was developed with a 30:70 ratio of paddy straw silage and commercial concentrate, where each BCM treatment was mixed with the concentrate. The details of each experimental diet and the chemical compositions of each feed ingredient on a dry matter (DM) basis can be found in Tables-1–3. The basal diet was formulated to meet the nutrient requirements of growing lambs to increase ADG above 100 g/head/day at about 3.5%–4% DMI (g/head/day) from BW [11]. Each feed refusal was weighed using a digital scale with an accuracy of 1 g (Henherr ACS-H1 LED) on the next morning to calculate DMI. Clean drinking water was provided *ad libitum*. Feed efficiency (%) was calculated by dividing ADG by DMI and multifield by 100%.

**Table-1:** Feed ingredients and their compositions (%DM basis) in the experimental diets.

Ingredients	Experimental diets			

	Control	BCM-5	BCM-10	BCM-15
Fermented paddy straw	30	30	30	30
Commercial concentrate	70	65	60	55
BCM	0	5	10	15

DM=Dry matter, BCM-5=Black cumin meal in concentrate at 5%, BCM-10=Black cumin meal in concentrate at 10%, BCM-15=Black cumin meal in concentrate at 15%

**Table-2:** Mean nutrient contents (%DM, or otherwise stated) of the feed ingredients (n = 2).

Nutrients	Paddy straw silage	Commercial concentrate	Black cumin meal
DM (% fresh sample)	31.7	93.5	94.9
Crude ash	21.0	10.2	6.64
Crude protein	7.33	14.7	36.8
Ether extract	2.04	4.88	5.55
NDFom	56.2	44.4	24.5
ADFom	38.2	28.0	15.5
Metabolizable energy (MJ/kg)	3.38	6.70	6.63
Total tannins	4.80	1.75	21.6

NDFom=Neutral detergent fibers (OM basis), ADFom=Acid detergent fibers (OM basis), DM=Dry matter, OM=organic matter

**Table-3:** Nutrient contents (%DM, or otherwise stated) of each concentrate treatment.

Nutrients	BCM-0	BCM-5	BCM-10	BCM-15
DM (% fresh sample)	73.9	74.0	74.1	74.1
Crude ash	13.5	13.3	13.1	12.9
Crude protein	12.5	13.6	14.7	15.8
Ether extract	4.03	4.06	4.10	4.13
NDFom	47.9	47.0	46.0	45.0
ADFom	31.0	30.4	29.8	29.2
Metabolizable energy (MJ/kg)	5.84	5.84	5.83	5.83

NDFom=Neutral detergent fibers (OM basis), ADFom=Acid detergent fibers (OM basis), DM=Dry matter, OM=Organic matter, BCM-5=Black cumin meal in concentrate at 5%, BCM-10=Black cumin meal in concentrate at 10%, BCM-15-Black cumin meal in concentrate at 15%

Rice straw silage was prepared by chopping fresh rice straw (5–10 cm long) using a chopper machine (PT. Adhika Raya, Indonesia). A molasses solution was also prepared by dissolving molasses in water at a 1:9 ratio. After that, chopped paddy straw was gradually placed into each blue plastic barrel (120 L capacity), and at the same time, molasses solution was evenly sprayed onto the straw. It was then to press the straw inside each barrel to make it as compact as possible and to maximize the oxygen-free condition. Each barrel was finally closed tightly with a lid and metal fastener. Paddy straw silage contained approximately 4% molasses solution. Commercial mixed concentrate (Starter grade, SG2) was purchased from a feed mill for PT. Dilar Lintas Raya (Tasikmalaya, Indonesia). The mixed concentrate consisted of palm kernel meal (21%), cassava meal (15%), coffee husk (11%), rice bran (10.5%), copra meal (9%), molasses (8.5%), dried cassava (8.45%), cacao husk (6%), soybean meal (2%), rapeseed meal (1.5%), distillers dried grain with soluble (1%), wheat pollard (0.5%), mineral and vitamin premix (Lagantor F1 Customix, Kalbe Animal Health, 2%), lime (2%), corn gluten feed (1%), sodium bicarbonate (0.35%), and salt (0.2%). In addition, BCM was collected from a PT feed mill. Baqara Muda Perkasa (Subang, Indonesia). BCM is a residual product from black cumin (*Habbatussauda*) oil fabrication in an herbal company (PT. Habbasyi Niaga Utama, Depok, Indonesia). BCM was grounded in a hammer mill before its use as a diet ingredient. Each diet ingredient was sent to the laboratory for chemical analysis, as described in the chemical analysis section below.

### Blood sampling

After 70 days of a feeding trial, approximately 3 mL blood samples were collected through the vena coccygeal of each experimental lamb before morning feeding, transferred into an ethylenediaminetetraacetic acid tube, placed in a cool box, and transported to the laboratory. After that, all blood samples were centrifuged (1107× *g*, 10 min) to collect blood plasma. Each plasma sample was placed in an Eppendorf tube and stored at −20°C until performing several analyses, such as total protein (g/dL, Biuret method) and albumin (g/dL, Bromeresol green method). Blood cholesterol (mg/dL) was determined using the cholesterol oxidase phenylperoxidase amino phenozonphenol method, whereas triglyceride (mg/dL) was determined using the glycerol-3-phosphate oxidase method, as described by Adriani *et al*. [12]. Blood glucose (mg/dL) was measured using Folin-Wu method, as described by Adriani *et al*. [13].

### Digestibility trial

After 14 days of adaptation, a 70-day feeding trial, and blood collection, the digestibility experiment was then conducted for 9 days (2 days adaptation and 7 days total fecal collection) using 24 heads of the same experimental lambs (six out of seven heads per treatment group were selected, n = 6). The procedure for total fecal collection and its measurements for the digestibility trial was as described by Ramdani *et al*. [11].

### Chemical analysis

During the study, feed samples were regularly collected from the corresponding storage bins for DM measurements. Each feed was sampled from different parts (the top, middle, and bottom) of the representative storage bins. Each feed or feces sample was dried in an oven at 58°C for 48 h. Each dried sample was grounded in a disk mill and passed through a 1-mm screen before being subjected to various nutrient determination. (DM, Association of Official Analytical Collaboration [AOAC] 934.01), ash (AOAC 924.05), (CP, AOAC 920.39), and ether extract (AOAC 920.39) were measured using the standard procedures of the AOAC [14]. The neutral detergent fibers (NDFom, without decalin and α-amylase) and acid detergent fibers (ADFom) were analyzed as described by Van Soest *et al*. [15]. Metabolizable energy (ME) was calculated using the procedure of Menke and Steingass, as described by Ramdani *et al*. [11]. Total tannin content was analyzed using the Folin–Ciocalteu method following the procedure of Makkar [16]. Most chemical compositions are expressed as % DM, except for DM (% fresh sample) and ME (MJ/kg DM).

### Statistical analysis

Each nutrient content of the feed ingredients was calculated as the average from a duplicate analysis (n = 2). The experimental data were statistically analyzed using one-way analysis of variance in the IBM SPSS 26.0 software (SPSS Institute, Chicago, IL, USA). The data showing significant differences at p < 0.05 were further analyzed using Duncan’s test to identify differences among treatments. The residual data were analyzed for normality by passing the Shapiro–Wilk test of normality at p > 0.05.

## Results

BCM is generally rich in protein and tannins but low in fiber fractions. According to Table-2, the quality of BCM was generally better than that of the concentrate. BCM had greater CP content, lower fiber fractions, and similar metabolizable energy content than the concentrate. Table-3 also shows the nutrient content of BCM-containing diets. Higher BCM supplementation in rice straw- and concentrate-based diets led to greater CP content in the diet.

**Table-4:** Mean BW (kg), ADG (g/head/day), DMI (g/head/day), and feed efficiency (%) of lambs supplemented with different levels of BCM (n = 7).

Parameters	Control	BCM-5	BCM-10	BCM-15	SEM	p-value
Initial BW	21.0	22.6	20.6	18.6	0.90	0.061
Final BW	30.1	32.4	33.0	29.1	1.08	0.124
ADG						
Day-35	126.1^a^	132.7^ab^	195.1^c^	180.0^bc^	15.3	0.012
Day-70	133.9	147.3	159.2	140.0	14.4	0.659
DMI						
Day-35	1.068	1.118	1.140	1.102	24.7	0.230
Day-70	1.189^a^	1.244^b^	1.260^b^	1.247^b^	14.0	0.012
Feed efficiency						
Day-35	11.7^a^	11.9^a^	17.2^b^	16.4^b^	1.44	0.023
Day-70	11.3	11.9	12.5	11.2	1.17	0.857

^a,b,c^Values in the same row with different letters showed significant differences (p < 0.05). Control=Black cumin meal in concentrate at 0%, BCM-5=Black cumin meal in concentrate at 5%, BCM-10=Black cumin meal in concentrate at 10%, BCM-15=Black cumin meal in concentrate at 15%, DMI=Dry matter intake, BW=Body weight, ADG=Average daily gain, SEM=Standard error of mean

Table-4 presents the means of BW, ADG, DMI, and feed efficiency of the experimental lambs during the 35 and 70 days of the feeding trial. The BCM-10 and BCM-15 treatments improved (p < 0.05) ADG and feed efficiency compared with the control treatment during the 35-day feeding trial. BCM seemed to be greater in palatability, as all BCM treatments had greater (p < 0.05) DMI compared with the control during the 70-day feeding trial. According to Tables-5 and 6, BCM-10 and BCM-15 had greater (p < 0.05) DM and organic matter (OM) digestibility and blood total protein. BCM-15 had the highest (p < 0.01) blood triglyceride content, whereas BCM-10 was likely to have higher (p < 0.1) blood glucose content among the other treatments. On the other hand, the data presented in Table-6 indicates that the use of BCM did not affect blood cholesterol and albumin.

**Table-5:** Mean %DM and %OM digestibility of lambs supplemented with different levels of BCM (n = 6).

Parameters	Control	BCM-5	BCM-10	BCM-15	SEM	p-value
DM	59.0^a^	62.4^ab^	68.0^b^	66.3^b^	2.03	0.047
OM	59.8^a^	63.7^ab^	69.0^b^	66.5^b^	1.86	0.026

^a,b,c^Values in the same row with different letters showed significant differences (p < 0.05). Control=Black cumin meal in concentrate at 0%, BCM-5=Black cumin meal in concentrate at 5%, BCM-10=Black cumin meal in concentrate at 10%, BCM-15=Black cumin meal in concentrate at 15%, DM=Dry matter, OM=Organic matter, SEM=Standard error of mean

**Table-6:** Mean blood cholesterol (mg/dL), triglyceride (mg/dL), glucose (mg/dL), total protein (g/dL), and albumin (g/dL) of lambs supplemented with different levels of BCM (n = 7).

Parameters	Control	BCM-5	BCM-10	BCM-15	SEM	p-value
Cholesterol	74.9	89.8	79.2	99.5	8.58	0.209
Triglyceride	78.4^a^	92.1^a^	94.3^a^	118.3^b^	3.98	0.001
Glucose	72.7	71.9	87.0	75.5	4.55	0.099
Total Protein	2.95^a^	3.83^ab^	4.09^b^	4.46^b^	0.29	0.013
Albumin	3.05	3.29	3.40	3.25	0.10	0.176

^a,b,c^Values in the same row with different letters showed significant differences (p < 0.05). Control=Black cumin meal in concentrate at 0%, BCM-5=Black cumin meal in concentrate at 5%, BCM-10=Black cumin meal in concentrate at 10%, BCM-15=Black cumin meal in concentrate at 15%. SEM=Standard error of mean

## Discussion

Table-2 shows that BCM contained 36.8% CP. Previous studies by Retnani *et al*. [7] and Mahmoud and Bendary [17] reported that the CP content in BCM ranged from 29.8% to 33.1%. An increase in BCM levels in the diet also leads to a corresponding increase in CP content (Table-3). The black cumin processing sector maintains a low temperature of a maximum 30°C during the extraction of black cumin seeds [7]. This condition can maintain the active substances and nutrient content of BCM without any damage.

Approximately 10% dietary BCM is the optimum level for increasing ADG in feedlot lambs, especially in the 1^st^ month of the fattening period. A similar trend was observed by Obeidat [18], who found that growing Awassi lambs had the highest ADG for a diet containing BCM compared with the control. BCM is rich in protein [17] and is, therefore, a good source of protein for growing lambs. Higher DMI resulted in greater nutrient intake, leading to increased ADG. Nutrients are used in the metabolic system of livestock to fulfill their basic life requirements and to increase BW. Dietary protein increases adipose tissue formation and ultimately increases BW [19]. Protein and energy have significantly contributed to increasing tissue mass [20].

In this study, improved ADG agreed with increased DMI, confirming that BCM is a palatable feed ingredient. Lambs are sensitive to the physical properties of feed ingredients. The sense of taste is very important because it plays a fundamental biological role in regulating feed intake in livestock [21]. Basically, the tannin content in BCM has a bitter taste and is less attractive to livestock, but this compound is also thought to provide an aroma that can increase palatability and stimulate lambs to consume the diet more frequently. According to Ramdani *et al*. [11], dietary green tea dust rich in tannins increased ADG in fattening lambs without any harmful effects on feed intake and nutrient digestibility. In an appropriate dose, dietary inclusion of tannins can improve the nutrient utilization efficiency of the diet, leading to an increase in muscle fiber size and skeletal muscle mass in lamb, reflecting improvements in DMI and ADG of lambs supplemented with tannins [22].

The feed efficiency is obtained by dividing ADG by DMI. Feed efficiency is an important factor in the farming industry, and it is related to several aspects, such as environmental, economic, and social [23]. Based on these results, dietary BCM can improve DMI, ADG, and feed efficiency. The high CP in BCM supports rumen microbes in degrading the diet utilized by livestock. Obeidat [18] reported that BCM was an efficient source of feed ingredients, significantly reducing feed costs. Similarly, Abdullah and Farghaly [24] reported that black cumin may modulate microorganisms in the rumen and their capabilities for digestion, resulting in more efficient digestion. According to Obeidat *et al*. [25], feed efficiency is influenced by increasing the nutrient content in the diet and increasing digestibility.

The data presented in Table-4 indicate that DM digestibility in the current study ranged from 59.0% to 68.0%. Abdullah and Farghaly [24] reported that BCM supplementation increases DM and OM digestibility in Farafra lambs. A previous meta-analysis confirmed that BCM supplementation contributed to greater DMI and nutrient digestibility, resulting in improved nutrient use efficiency [10]. BCM also contains high-quality CPs that can be digested easily [7]. Increased protein and BCM content in a diet is helpful for rumen microbe activity. Barkah *et al*. [26] reported that BCM was a protein source that significantly affected ammonia (NH_3_) production in the rumen, which is then used by rumen microbes for microbial protein synthesis and increased digestibility. This study found that dietary BCM might modulate microorganisms and increase the digestion of rice straw-based diet in the rumen. Rumen-degraded protein will be directly utilized by the lamb’s body. Therefore, proteins degraded by rumen microbes are converted into NH_3_ that is further used by rumen microbes to grow, especially cellulolytic bacteria. Cellulolytic bacteria can develop in a situation of high NH_3_ levels from the fermentation of protein in the diet, resulting in an increased ability to break down crude fiber (cellulose) [27]. As is widely recognized, the largest component of ruminant diets is carbohydrates, particularly crude fiber.

Dietary BCM in the typical diet of the current study also reduced NDF and ADF content (Table-3). Ma *et al*. [28] reported that the digestibility of a diet containing rice straw decreased with increasing levels of NDF and ADF. NDF and ADF are fiber fractions that include lignin, a substance that naturally binds cellulose and hemicellulose. This binding reduces the digestibility coefficient of diets by slowing down the digestion rate in the digestive tract [29]. The composition of fibers such as ADF and NDF may also influence behavioral activities, especially the time for rumination [30]. By reducing fiber fractions with dietary BCM supplementation, the diet could be more quickly digested.

In the current study, Dietary BCM did not affect blood cholesterol and albumin levels, but it increased blood triglyceride, glucose, and total protein levels in lambs. Similarly, Mustafa *et al*. [31] reported no significant effect of black cumin supplementation on cholesterol and albumin concentrations in Karadi lambs. In contrast, previous studies by Mohammed and Al-Suwaiegh [32] and Cherif *et al*. [33] reported that black cumin can reduce blood triglyceride levels in mammals. Cherif *et al*. [33] reported increased blood triglyceride levels due to dietary black cumin seed, which has a high content of unsaturated fatty acids, particularly linolic acid. The blood triglyceride level is influenced by high fat intake [7]. The higher concentration of total protein in dietary BCM could be attributed to increased hepatic function [32]. This condition could also be suspected based on the increased total protein in the blood along with the increased DM and OM digestibility in this study. Zanouny *et al*. [34] reported that black cumin supplementation in a lamb diet influenced the total protein in the blood and was expressed in the percentage of digestibility as well. Blood glucose levels in this study ranged from 71.9 to 87.0 mg/dL. According to Kaneko and Cornelius [35], blood glucose concentrations typically range from 50 to 100 mg/dL. The increased energy consumption as a result of higher DMI leads to increased consumption of carbohydrates, which are digested by glycolytic enzymes and converted into glucose. Blood glucose levels are controlled by insulin and glucagon, maintaining their levels stable [7].

## Conclusion

BCM can partially substitute for a commercial concentrate, leading to improved overall quality of rice straw-based diet, growth performance, protein and energy absorption in the blood, and nutrient digestibility during intensive fattening of Garut lambs without any detrimental effects. However, the advantageous effects of BCM as a feed supplement on ruminants appear to be dose-dependent. Approximately 10% of the BCM content in sheep diets is suggested to be the optimum level. Further studies are required to evaluate the bioactive components of BCM and their effects on reproductive, therapeutic, and anthelmintic parameters in ruminants.

## Authors’ Contributions

DR: Conceptualization, supervision, experimental design, and manuscript review and editing. KNJ: Conducted the study, data collection and statistical analysis, and manuscript preparation. IH and KRGA: Conceptualization, supervision, literature search, and, review and editing of the manuscript.: All authors have read, reviewed, and approved the final manuscript.
